# Acute effects of solar particle event radiation

**DOI:** 10.1093/jrr/rrt196

**Published:** 2014-03

**Authors:** Ann R. Kennedy, Drew Weissman, Jenine K. Sanzari, Gabriel S. Krigsfeld, X. Steven Wan, Ana L. Romero-Weaver, Eric S. Diffenderfer, L. Lin, K. Cengel

**Affiliations:** Perelman School of Medicine, University of Pennsylvania, Philadelphia, PA, USA

## Abstract

A major solar particle event (SPE) may place astronauts at significant risk for the acute radiation syndrome (ARS), which may be exacerbated when combined with other space flight stressors, such that the mission or crew health may be compromised. The National Space Biomedical Research Institute (NSBRI) Center of Acute Radiation Research (CARR) is focused on the assessment of risks of adverse biological effects related to the ARS in animals exposed to space flight stressors combined with the types of radiation expected during an SPE. The CARR studies are focused on the adverse biological effects resulting from exposure to the types of radiation, at the appropriate energies, doses and dose-rates, present during an SPE (and standard reference radiations: gamma rays or electrons). All animal studies described have been approved by the University of PA IACUC. Some conclusions from recent CARR investigations are as follows: (i) the relative biological effectiveness (RBE) values for SPE-like protons compared with standard reference radiations (gammas or electrons) for white blood cells (WBCs) vary greatly between mice, ferrets and pigs, with the RBE values being greater in ferrets than those in mice, and considerably greater in pigs compared with those in ferrets or mice [1, 2]. This trend for the data suggests that the RBE values for WBCs in humans could be considerably greater than those observed in small mammals, and SPE proton radiation may be far more hazardous to humans than previously estimated from small animal studies. (ii) Very low doses of SPE proton radiation (25 cGy) increase blood clotting times in ferrets, and the low SPE-like dose rate has more severe effects than high dose rate radiation [3]. (iii) Results from pig and ferret studies suggest that disseminated intravascular coagulation is a major cause of death at doses near the LD_50_ level for SPE-like proton and gamma radiation. (iv) Exposure to SPE-like proton or gamma radiation, in combination with simulated microgravity (hindlimb suspension), leads to a very high level of morbidity/mortality in mice given a bacterial challenge with non-toxic levels of *Pseudomonas aeruginosa* or *Klebsiella pneumoniae*; the threshold for this effect was 1.5 Gy. (v) T-cell activation was reduced in mice exposed to SPE-like radiation with or without simulated hypogravity (either partial weight suspension or hindlimb suspension) (e.g. [4]). (vi) Radiation and simulated hypogravity had synergistic effects on immune system biological endpoints (e.g. [5]). (vii) Pigs exposed to simulated SPE radiation exhibited increases in intracranial pressure that remained elevated over the 90-day experimental period. (viii) A major sparing effect of SPE-like low dose rate radiation (compared with the results for high dose rate radiation) was observed for ferret emesis parameters, such that the differences between the results for ferret exposure to low dose rate radiation (50 cGy/h) and controls were not statistically significant (for doses up to 2 Gy). For high dose rate SPE proton radiation, the threshold value for retching was 75 cGy, and for ferret vomiting, it was 1 Gy.
